# Recovery of the Total Birth Brachial Plexus Palsy without Surgical Treatment: A Single-Center, Retrospective Study and Literature Review

**DOI:** 10.1055/a-2321-0468

**Published:** 2024-06-10

**Authors:** Chaiyos Vinitpairot, Surut Jianmongkol

**Affiliations:** 1 Department of Orthopedics, Hand and Reconstructive Unit, Faculty of Medicine, Khon Kaen University, Khon Kaen, Thailand

**Keywords:** birth brachial plexus palsy, spontaneous recovery, global palsy, natural history

## Abstract

**Background** Spontaneous recovery of a birth brachial plexus disorder is difficult to predict. Although root avulsion and total plexus injury is indicated for surgical management, early nerve surgery is still doubtful. Hand motion is obviously an important indicator for predicting the function of an affected limb. However, the timing for diagnosing a transient or true total plexus injury from hand recovery is controversial. This study aimed to report the recovery time of total birth brachial plexus palsy in patients who did not undergo surgery due to various reasons.

**Methods** In this 15-year retrospective chart review, 45 patients of total birth brachial plexus injury with a mean follow-up time of 34.5 months, were included. Although patients met surgical indications, surgical management was abandoned for a variety of reasons. Imaging was not performed routinely and, nerve conduction study and Horner's syndrome were not consistently recorded in the past. All patients were evaluated for clinical improvement by motor power grading. The recovery time was reported as the median and interquartile range.

**Results** Forty-five patients were diagnosed with total birth brachial plexus injury. Out of 45 patients, 36 showed clinical evidence of recovering their hand motion within a median of 3 months. The median time for the recovery of elbow flexion and shoulder abduction was 4 months. The median for achieving antigravity or full motion recovery of elbow flexion, shoulder abduction, and hand flexion were 10, 10.5, and 7 months, respectively.

**Conclusion** In this study, spontaneous recovery of shoulder, elbow and hand motion substantially occurred in the patient diagnosed with a total birth brachial plexus palsy. True total plexus palsy can be distinguished from transient palsy by the recovery of hand motion at 3 months. Most of the patients, who had spontaneous recovery, potentially achieved antigravity or full hand movement without surgery.

## Introduction

Obstetric brachial plexus palsy (OBPI) typically occurs as a result of a challenging childbirth. Its incidence is approximately 0.3 to 0.4 in 1,000 infants. 1 2 The injury is caused by excessive traction on the shoulder during a difficult delivery. However, some reports argue that obstetric brachial plexus injury can happen not only during a vaginal delivery but also during a cesarean section. 3 4 Biceps muscle strength generally recovers within the first 3 months of life, which is considered a positive sign for good outcomes. A classic study by Gilbert and Tassin proposed that if biceps motor function recovers before 3 months of age, there will likely be positive functional outcomes after 5 years. 5 Subsequent research by other authors has indicated that spontaneous recovery can occur between 4 and 9 months after delivery. 6 7 8

The controversy amid the total birth brachial plexus injury also involve the time frame and the surgical outcome. Generally, the surgical reconstruction has been advocated to patient without hand recovery around 2 to 4 months but there was an evidenced reporting favorable outcome with surgery even at 6 months. 7 9 10 11 12 Additionally, the hand functional outcome may not be different between the surgical and nonsurgical patients. 13 Horner's sign serves as a surrogate of root avulsion and one of the indications for surgery, however this concept has been challenged by several authors. 14 15 16 17 18 Despite growing evidence of surgical outcome, the appropriate timing for observation in birth brachial plexus injury patients remains controversial due to limited evidence, especially the total plexus injury. The existing body of evidence regarding early surgery for total plexus injuries continues to expand, yet determining the precise timing for recovering of total plexus injury remains unclear. 4 Regarding the previous studies, early surgery also did not result in better outcome than late surgery and many patients were operated upon unnecessarily. 6 19

Despite the knowledge of microsurgical reconstruction in birth brachial plexus injuries, the effectiveness of surgical treatment compared to conservative approaches remains uncertain. Recent meta-analyses have failed to establish whether surgical intervention yields better outcomes. Inconclusive findings can be attributed to variations in functional outcomes and procedures among different studies, leaving this topic open to ongoing debate. 12 Nerve repair, nerve grafting, and nerve transfer are among the available options for reconstructing birth brachial plexus disorders. However, there is a lack of robust evidence regarding the optimal procedure and timing. While numerous reports exist on surgical outcomes, there is a scarcity of information regarding the outcomes of patients who have not undergone surgery. 20 21 Additionally, reports on spontaneous recovery of hand motor function are rare.

Our objective is to present the motor recovery and recovery time of total birth brachial plexus injury in patients who have not undergone surgical intervention.

## Methods

The study has been reviewed and approved by the University Ethics Committee for Human Research based on the Declaration of Helsinki and the ICH Good Clinical Practice Guidelines. The IRB approval number is HE641462. Forty-five charts, which were diagnosed as total birth brachial plexus disorder and did not undergo surgery between 2006 and 2021, were enrolled in this study. The diagnosis of birth brachial plexus disorder was based on an examination of referral documents and medical history in the charts. Patients who had undergone surgery, were referred after their motor power had reached the antigravity grade, upper arm type including Narakas classification 1 and 2, or had traumatic brachial plexus disorder were excluded from this study.

In our practice, the patients were monitored monthly until their initial motor power (M1) recovered. After the initial motor recovery, follow-up appointments were scheduled every 2 months to evaluate motor improvement until antigravity power was achieved.

The authors reviewed all charts and extracted relevant information such as birth weight, involvement of brachial plexus injury, motor power, and age of recovery. Motor power was evaluated and recorded using the grading system developed by Gilbert and Tassin, with adjustments made to the motor power assessment of the hand, as outlined in Table 1 . 22 The outcomes, including birth weight, length of follow-up, and motor power grade, were reported using the median and interquartile range. Age was reported in months. The initial recovery was the time that data of grade 1 motor recovery (M1) were first available in chart. The M1 motor grade was determined by any discernible contraction or tone of the muscle and minimal, albeit futile, motion. For the elbow, the humerus was immobilized and allowed free movement of the forearm. The biceps muscle, palpable beneath the skin, facilitated the physician in evaluating any movement or tone of the muscle belly. During shoulder examination, the patient lay on the table, and the physician stabilized the scapula and clavicle to prevent motion from the trapezius. The arm was left free for movement. If no visible movement was observed, the tone and palpation of the arm were assessed next. Generally, M1 motor power was assigned for the shoulder with resistance tone or slight movement of the arm—noticeable but functionally useless. Assessing the hand was proven challenging due to the inability to palpate the muscle belly. Determining the M1 of the hand relied on any resistance tone during flexion or slight motion that was just perceptible but futile. In the same way, antigravity recovery was obtained from the first grade 3 (M3) data available in the chart. The availability of data was summarized in Table 2 since we did not have a good pattern of data record in the past, so some data were not complete.

**Table 1:** Modified definition of motor power grading

Motor power grade	Definition
Grade M0	No movement, no muscle contraction
Grade M1	Muscle contraction, no movement
Grade M2	Movement without antigravity of shoulder, either abduction or forward flexion, elbow flexion and partial motion of hand
Grade M3	Antigravity movement of shoulder and elbow either abduction or forward flexion or full motion of hand

**Table 2:** Summary of the recovery data

Type	*N*	Median (IQR; months)
Total patients	45	
Grade M1 recovery		
Elbow flexion	42	4 (3–6.75)
Shoulder	42	4 (3–7)
Hand	35	3 (2–5.5)
Grade M3 recovery		
Elbow flexion	36	10 (5.75–19.25)
Shoulder	32	10.5 (6–16.75)
Hand	36	7 (4–13)

Abbreviation: IQR, interquartile range.

## Results

The mean follow-up time in this study was 34.5 months, ranging from 3 to 113 months. The mean birth weight of the patients was 3,750 g, ranging from 2,590 to 5,250 g. Out of 45 patients who were diagnosed with total plexus injury, data on the M1 recovery of elbow flexion, shoulder movement, and hand motion were available for 42, 42, and 35 patients, respectively. Data on the M3 recovery of elbow flexion, shoulder movement, and hand motion were available for 36, 32, and 36 patients, respectively. The availability of hand recovery data is summarized in Table 3 .

**Table 3:** The summary of availability of hand recovery data

M1 recovery M3 recovery	Available	Unavailable
Available	32	5
Unavailable	3	5

Regarding hand recovery, out of five patients who had no recovery data for both M1 and M3, three patients did not regain hand motion, and one patient had only grade M2 recovery data, though the exact time was unclear. The remaining patient, who lacked hand recovery data, was lost to follow-up at 7 months.

There were five patients who had no data on M1 hand recovery but had M3 recovery. All of them had regained hand function before visiting our hospital, and the timing was not recorded. As for the other three patients who had only M1 recovery data, they did not reach grade M3 recovery, so the data were not provided. In summary, 41 patients achieved a recovery of at least grade M1 for their hand, and 37 patients reached a recovery level of grade M3 for their hand.

Two patients did not have a record of elbow recovery; however, one of the patient was reached by phone. This patient eventually regained grade M2 shoulder power at some point. In summary, 44 out of 45 patients had regained grade M1 elbow recovery, and 36 patients spontaneously recovered their elbow function up to grade M3. One patient was lost to follow-up before shoulder recovery was assessed. In summary, grade M1 shoulder recovery was observed in 42 patients, and grade M3 was seen in 32 out of the 45 patients.

**Fig. 1:**
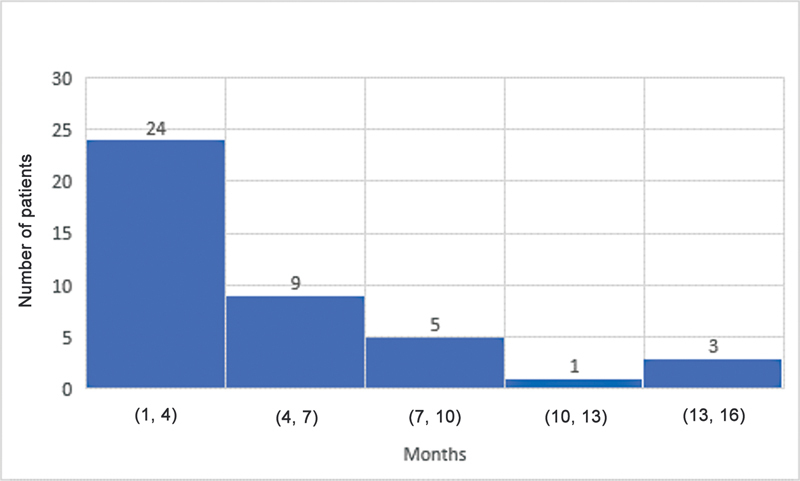
Grade M1 recovery of elbow flexion. M1, grade 1 motor recovery.

The median ages for grade M1 recovery of elbow flexion, shoulder abduction, and hand movement were 4, 4, and 3 months, respectively. Elbow flexion and the shoulder both reached antigravity (M3) power at a median age of 10 and 10.5 months, respectively. The median age for grade M3 hand recovery was 7 months. The results for shoulder, elbow, and hand recovery have been summarized in Table 2 and Figs. 1 2 3 4 5 6 .

**Fig. 2:**
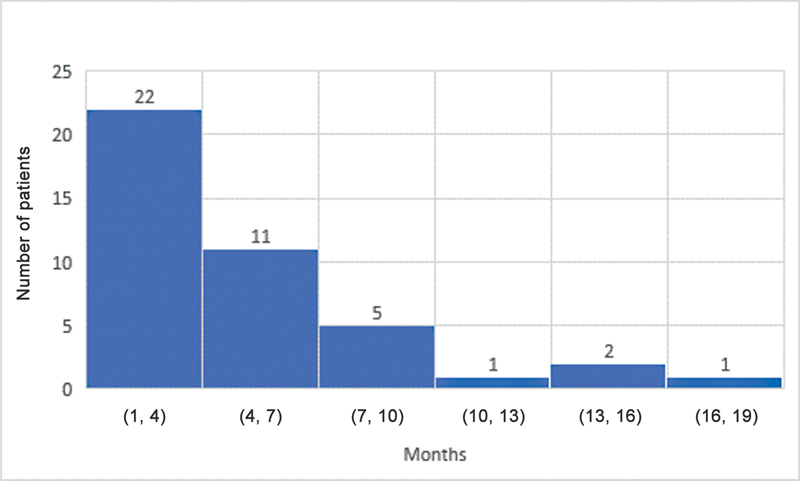
Grade M1 recovery of shoulder motion. M1, grade 1 motor recovery.

**Fig. 3:**
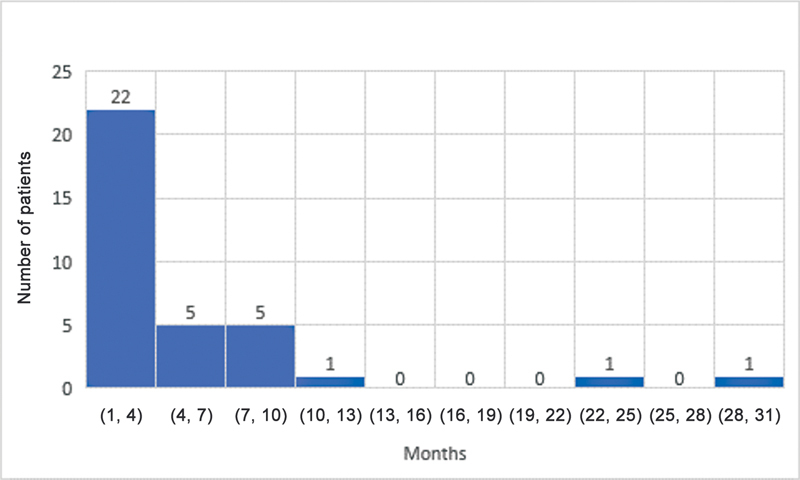
Grade M1 recovery of hand motion. M1, grade 1 motor recovery.

**Fig. 4:**
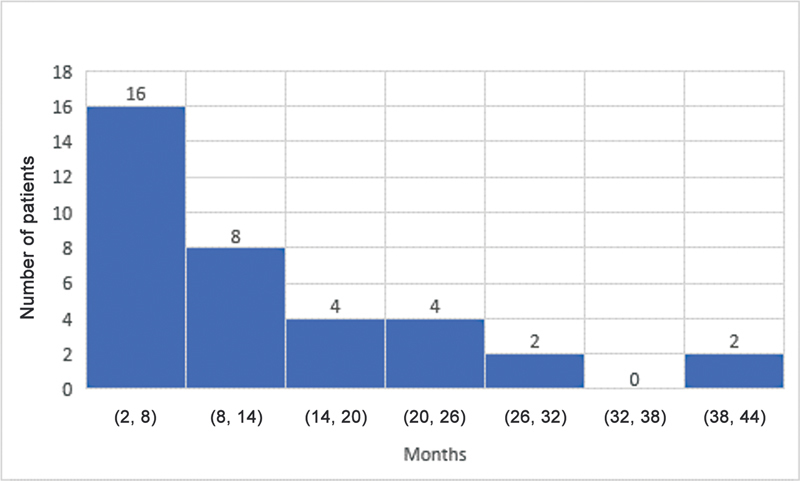
Grade M3 recovery of elbow flexion. M3, grade 3 motor recovery.

**Fig. 5:**
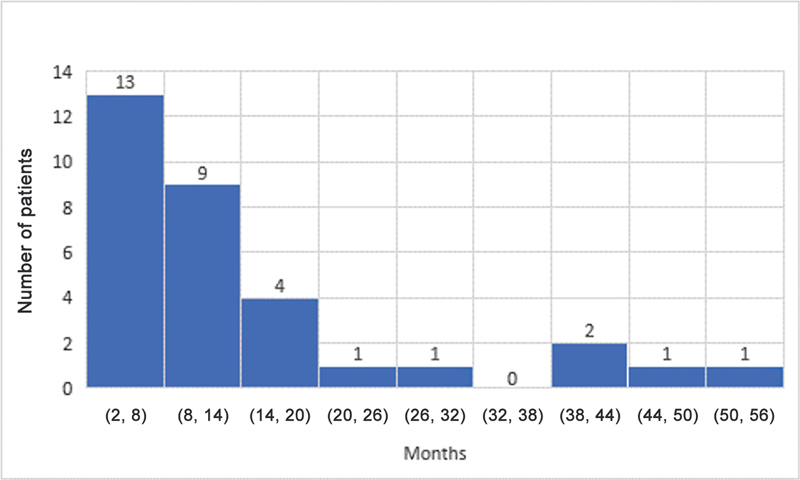
Grade M3 recovery of shoulder motion. M3, grade 3 motor recovery.

**Fig. 6:**
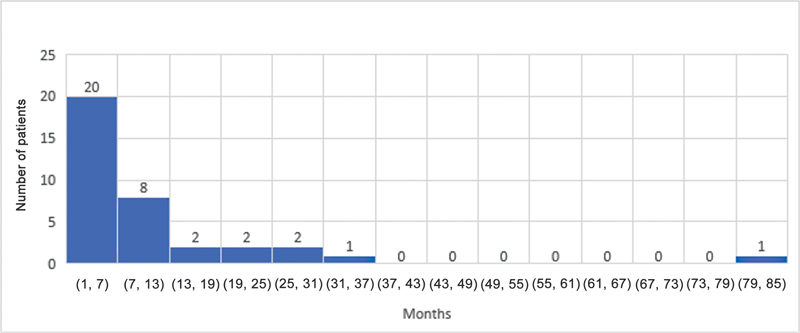
Grade M3 recovery of hand motion. M3, grade 3 motor recovery.

## Discussion

The natural history of muscle strength recovery has been described in previous reports, but most of the evidence comes from retrospective reviews. Due to the rarity of the injury and legal issues, conducting a prospective randomized study comparing surgical and conservative treatments is challenging. In a classic study by Gilbert and Tassin, it was reported that patients without biceps recovery within 3 months should undergo microsurgical reconstruction to achieve good shoulder function. 5 Some authors have considered 6 months of age without recovery as an indication for surgery, while others, like Clark and Curtis, 8 used 9 months.

Subsequently, there has been limited evidence demonstrating muscle strength recovery without microsurgical reconstruction. 6 8 13 23 Hems et al reported that nearly all their infants with birth brachial plexus palsy were able to reach their mouths without surgery. 23 Similarly, in our study, we found that the median time for the initial recovery of elbow, shoulder, and hand was 4, 4, and 3 months, respectively, and that most of them achieved grade M3 recovery. The mean recovery time in our study was consistent with previous studies, mostly occurring between 3 and 6 months. 6 8 13 23 Nevertheless, this study added the data of hand recovery because patients in our study were total plexus injury.

The controversial issue is determining when the surgeon should diagnose true or transient total plexus injury. Birch et al reported 2 weeks postdelivery, but some may argue otherwise. 4 24 In our study, we noticed the recovery of hand at first month only in six patients. The median time for hand recovery in this study was 3 months, leading us to believe that these patients do not have true total plexus injury but rather a transient conduction block. Therefore, we propose that waiting until 3 months is worthwhile before diagnosing the true or transient total plexus palsy.

One of the controversial points is the potential benefit of early surgery, but proving this with strong evidence is challenging due to the heterogeneity of outcome measures in studies on birth brachial plexus palsy. Some studies focus on motor outcome measures, while others emphasize functional outcomes, leading to variations in the types of functional assessments and motor grading systems used among these studies. On one hand, several authors advocate for early surgery, particularly in cases of total plexus palsy. Haerle and Gilbert reported that 75% of their 73 operative patients achieved useful hand function (Gilbert and Ramondi's hand classification grade 3–5) after 8 years of follow-up. 9 They recommended surgery for patients with Horner's syndrome who did not regain hand capability within 3 months. In the study by Terzis and Kokkalis, all six total plexus palsy infants underwent primary reconstruction before 3 months and did not require a secondary surgery. Forty-six out of 61 infants achieved a grade 4 Gilbert–Ramondi hand scale or higher after surgery. 10 Birch et al reported recovery in 33 out of 80 patients who underwent nerve reconstruction at 2 months. Fifty-seven percent of their reconstructed patients achieved Ramondi's grade 4 or higher. 24 In the article by Pondaag and Malessy, 15 patients with useful hand function were reconstructed by either nerve grafting or nerve transfer within 4.4 months. 25 Maillet and Romana also reported a satisfactory outcome of hand function in early nerve surgery for total plexus injury with a mean follow-up of 7 years and 10 months, although hand recovery may not be as good as shoulder and elbow recovery. 26

On the other hand, a study by Kirjavainen et al demonstrated only a 2.16 point increase in the Gilbert–Ramondi score for patients with total plexus palsy who underwent surgery. 27 In the study by Smith et al, most patients achieved useful hand function even with surgery at 6 months. 7 In the case series by Störmbeck et al, they observed literal sensation recovery but incomplete recovery of hand motor function in operative patients. 13 The outcome of operative patients was not significantly different from nonoperative patients in terms of hand function. Dumont et al published results of 20 operative patients, with most of them managed by neurolysis of their lower trunk. Their study showed that total plexus palsy infants with some preservation of hand movement before the operation had better hand movement scores than those with no prior recovery. Additionally, 17 out of 20 infants who had no recovery before surgery scored between 0.3 and 3.8 in hand movement, indicating that no patient achieved antigravity movement and satisfactory grasp. 11 Our study provided data on patients who did not undergo surgery and recovered naturally. Despite the grade M3 recovery, many patients in this study required late reconstruction surgery such as tendon transfers and denotational osteotomy and the functional outcome has not been recorded. Therefore, the function of affected limb could not compare with previous studies.

There is a growing debate among neurosurgeons regarding the spontaneous recovery of elbow and shoulder function, but hand recovery is considered more crucial. The specific timing to diagnose the true total plexus type and determine absence of hand recovery is controversial. Some patients may regain their hand motion a few months after delivery, but the actual data on this issue are limited. Performing early surgery in such cases may lead to unnecessary surgical interventions.

The early surgery for Horner's syndrome in birth brachial plexus injury has been questioned by several authors. 14 15 16 18 Although it is a strong indicator of root avulsion in adult brachial plexus palsy, the correlation between Horner's syndrome and the outcome of birth brachial plexus injury is currently controversial. Chuang et al found that Horner's sign was not a reliable predictor of poor prognosis in babies with a birth weight of less than 4 kg. The relationship between hand function sometimes does not correlate with Horner's sign. 15 16 Birch also reported in their series that Horner's syndrome was not always a sign of poor prognosis and hopelessness. 14 Subsequently, there was a reported histopathological difference between Horner's syndrome in obstetric and adult brachial plexus palsy. Huang et al described the innervation of the sympathetic ganglion on the C7 ventral root in babies but not in adult cadavers. The avulsion of C7 may cause Horner's syndrome, while the C8 and T1 roots were not avulsed. Infants with total plexus palsy and Horner's syndrome sometimes recovered hand function, or the C8 and T1 roots were found to be intact intraoperatively. 17 Recently, a study on total obstetric plexus injury by Yoshida and Kawabata has shown that Horner's syndrome has no prognostic value for predicting a poor outcome. 18 In contrast, Al-Qattan et al reported a poor prognosis for the spontaneous recovery of total obstetric brachial plexus injury patients who had Horner's syndrome, along with El-Sayed, who also reported poor spontaneous recovery in cases of concurrent Horner's syndrome involving C6 and C7 in extended Erb obstetric brachial plexus injuries. 28 29

Uncertainty surrounded the data on Horner's syndrome, making it impossible to differentiate between grade 3 and 4 of the Narakas classification in our study. The study did not report wrist recovery time and nerve conduction since these variables were inconsistently recorded. Moreover, our records lacked comprehensive descriptions of elbow and shoulder contracture. Our indication for surgery was the lack of recovery in biceps muscle power within 3 months, Horner's syndrome, or total plexus palsy. However, we faced limitations as a well-trained hand surgeon specializing in birth brachial plexus was not available in the past, and some parents were against advice of surgery mainly because of the partial recovery and the risk of surgery on their infants. Additionally, due to financial limitations in our poor area in developing country, many parents could not afford the travel seeking the specialized medical treatment, and at the time we did not have well-trained hand surgeon. Magnetic resonance imaging (MRI) and computed tomography (CT) myelograms were not routinely conducted for this study due to the long waiting list for imaging and concerns regarding the poor quality of our MRI machine in the past.

The authors do not oppose the current practice of recommending surgical reconstruction for total plexus palsy, as most cases are caused by avulsion of C8 and T1 roots, 52% of 51 patients in the study by Terzis and Kokkalis. 10 However, considering a 3-month observation period to classify patients as true or transient total plexus injury may be beneficial. This study suggests that patients initially diagnosed with total plexus palsy may require a few months before a definitive diagnosis can be made and surgery can be considered. Stronger evidence is needed to determine the ideal timing for observation, and the risks and benefits should be thoroughly discussed with parents. Performing surgery before 3 months of age poses challenges related to anesthesia, intraoperative care, and postoperative management. Moreover, complications such as phrenic nerve injury, thoracic duct injury, vascular injury, accidental extubation, and wound infection have been reported at rates as high as 33.5 to 50%. 30 31 Apart from patient-related factors, a recent study showed that microsurgical intervention in 3-month-old infants costs more than twice as much as in 6-month-old infants. 32

As a retrospective study, this study has several limitations. Firstly, the availability of data is a main problem of this study since we did not have a good pattern of recording the brachial plexus data in the past. In our study, missing data refer to the unavailability of the initial recovery (M1) or antigravity (M3) recovery data, making them indeterminable. The absence of M1 records may be due to various reasons, such as no motor recovery, recovery occurring before the patient's referral to our hospital, loss of follow-up before the initial recovery assessment, or simply no recorded information. The absence of M3 records can result from factors like loss of follow-up before reaching M3 recovery, the absence of M3 recovery, or a lack of data even if recovery occurred. Given the retrospective nature of our study, we made efforts to collect data through chart reviews and phone calls. Unfortunately, there are instances where we could not retrieve several pieces of data. The data were inconsistently recorded, especially concerning the nerve conduction study, wrist motion, and Horner's syndrome. However, we believe that the available data can represent the timing of the patient that is capable of recovery without surgery. Secondly, although the majority of patients were initially diagnosed with total plexus palsy by non-neurosurgeons, the paralyzed hand is often visibly obvious and involves the affected part. Either the parents or the physician could recognize the hand weakness and document it during the history-taking process. Thirdly, even the recovery rate is quite high in our study but neither functional outcomes nor sensations were assessed. While functional outcomes at a specified childhood age could have been a good predictor for past treatment, it was challenging to do so in this study due to a loss of contact with many patients. Finally, imaging was not routinely performed in this study due to the reasons mentioned.

In conclusion, most total birth brachial plexus injury patients who experienced initial recovery of elbow, shoulder, and hand can achieve antigravity or full motion grade. Hand recovery could occur spontaneously at 3 months, therefore a diagnosis of transient or true total plexus injury is recommended not earlier than 3 months after delivery.
